# Relationship between flexion of the neck and changes in intracranial pressure

**DOI:** 10.1186/2045-8118-12-S1-P39

**Published:** 2015-09-18

**Authors:** Sarah Skovlunde Hornshoej Pedersen, Morten Andresen, Alexander Lilja Jørgensen, Dorte Harpsoee Christoffersen, Marianne Juhler

**Affiliations:** 1 CSF Copenhagen Studygroup, Denmark

## Introduction

Intracranial pressure (ICP) monitoring at our department includes a standardized postural change examination to evaluate its effects on ICP. During these procedures we observed that neck flexion caused an increase in ICP in one patient. To clarify if the observation was a random occurrence or if it could be reproduced, we added neck flexion and extension to the standard examination.

## Methods

All patients undergoing invasive ICP monitoring at our department were included prospectively. The postural change examination consists of eight standard postures including both horizontal and vertical positions. In this abstract we focus specifically on the effect of neck flexion on ICP in the vertical positions. We examined the effect on ICP with the patient sitting upright with a straight back and 1) a straight neck or 2) maximal neck flexion, and the patient sitting bent in “lumbar puncture position” with 3) neck flexion or 4) a straight neck. Each posture was maintained for ten minutes. We recorded ICP and demographic data.

## Results

All 45 patients completed both measurements in the upright sitting position (18 male), while 38/45 patients also completed both measurements in “lumbar puncture position” (16 male). In both positions, flexion of the neck caused an increase in ICP in all patients.

In the upright sitting position the median ICP with 1) a straight neck was –5 mmHg (range –28 to 8 mmHg) while the median ICP with 2) neck flexion was 3 mmHg (range –18 to 19 mmHg). The median increase in ICP when flexing the neck was 8.5 mmHg (range 2.4 to 18 mmHg). The increase was highly significant (p < 0.001).

The median ICP in the lumbar puncture position was 13 mmHg (range –15 to 38 mmHg) and -2.7 mmHg (range –28 to 14 mmHg) for respectively 3) neck flexion and 4) a straight neck. The median increase was 15.89 mmHg (range 8 to 25.7 mmHg). This increase in ICP was likewise highly significant (p < 0.001).

## Conclusion

The results indicate that the position of the neck has a more important influence on ICP than previously presumed. We speculate that the increase in ICP is a result of either compression of the jugular veins or the vertebral canal.

Further investigation indicates that the jugular veins indeed are affected by the position of the neck. 10 patients undergoing invasive ICP monitoring and postural change examination have been examined by ultrasound (UL). Primary results shows compression of the jugular veins in response to neck flexion and a correlation with an increase in ICP.

